# SMDB: a Spatial Multimodal Data Browser

**DOI:** 10.1093/nar/gkad413

**Published:** 2023-05-22

**Authors:** Ruifang Cao, Yunchao Ling, Jiayue Meng, Ao Jiang, Ruijin Luo, Qinwen He, Anan Li, Yujie Chen, Zoutao Zhang, Feng Liu, Yixue Li, Guoqing Zhang

**Affiliations:** National Genomics Data Center& Bio-Med Big Data Center, CAS Key Laboratory of Computational Biology, Shanghai Institute of Nutrition and Health, University of Chinese Academy of Sciences, Chinese Academy of Science, Shanghai 200031, China; National Genomics Data Center& Bio-Med Big Data Center, CAS Key Laboratory of Computational Biology, Shanghai Institute of Nutrition and Health, University of Chinese Academy of Sciences, Chinese Academy of Science, Shanghai 200031, China; National Genomics Data Center& Bio-Med Big Data Center, CAS Key Laboratory of Computational Biology, Shanghai Institute of Nutrition and Health, University of Chinese Academy of Sciences, Chinese Academy of Science, Shanghai 200031, China; School of Computer Science, Wuhan University, Wuhan 430072, China; Shanghai Southgene Technology Co., Ltd., Shanghai 201203, China; National Genomics Data Center& Bio-Med Big Data Center, CAS Key Laboratory of Computational Biology, Shanghai Institute of Nutrition and Health, University of Chinese Academy of Sciences, Chinese Academy of Science, Shanghai 200031, China; Britton Chance Center for Biomedical Photonics, Wuhan National Laboratory for Optoelectronics, MoE Key Laboratory for Biomedical Photonics, Huazhong University of Science and Technology, Wuhan 430074, China; HUST-Suzhou Institute for Brainsmatics, JITRI Institute for Brainsmatics, Suzhou 215123, China; National Genomics Data Center& Bio-Med Big Data Center, CAS Key Laboratory of Computational Biology, Shanghai Institute of Nutrition and Health, University of Chinese Academy of Sciences, Chinese Academy of Science, Shanghai 200031, China; Britton Chance Center for Biomedical Photonics, Wuhan National Laboratory for Optoelectronics, MoE Key Laboratory for Biomedical Photonics, Huazhong University of Science and Technology, Wuhan 430074, China; School of Computer Science, Wuhan University, Wuhan 430072, China; National Genomics Data Center& Bio-Med Big Data Center, CAS Key Laboratory of Computational Biology, Shanghai Institute of Nutrition and Health, University of Chinese Academy of Sciences, Chinese Academy of Science, Shanghai 200031, China; Guangzhou Laboratory, Guangzhou 510005, China; National Genomics Data Center& Bio-Med Big Data Center, CAS Key Laboratory of Computational Biology, Shanghai Institute of Nutrition and Health, University of Chinese Academy of Sciences, Chinese Academy of Science, Shanghai 200031, China

## Abstract

Understanding the relationship between fine-scale spatial organization and biological function necessitates a tool that effectively combines spatial positions, morphological information, and spatial transcriptomics (ST) data. We introduce the Spatial Multimodal Data Browser (SMDB, https://www.biosino.org/smdb), a robust visualization web service for interactively exploring ST data. By integrating multimodal data, such as hematoxylin and eosin (H&E) images, gene expression-based molecular clusters, and more, SMDB facilitates the analysis of tissue composition through the dissociation of two-dimensional (2D) sections and the identification of gene expression-profiled boundaries. In a digital three-dimensional (3D) space, SMDB allows researchers to reconstruct morphology visualizations based on manually filtered spots or expand anatomical structures using high-resolution molecular subtypes. To enhance user experience, it offers customizable workspaces for interactive exploration of ST spots in tissues, providing features like smooth zooming, panning, 360-degree rotation in 3D and adjustable spot scaling. SMDB is particularly valuable in neuroscience and spatial histology studies, as it incorporates Allen's mouse brain anatomy atlas for reference in morphological research. This powerful tool provides a comprehensive and efficient solution for examining the intricate relationships between spatial morphology, and biological function in various tissues.

## Graphical Abstract

**Graphical Abstract:**
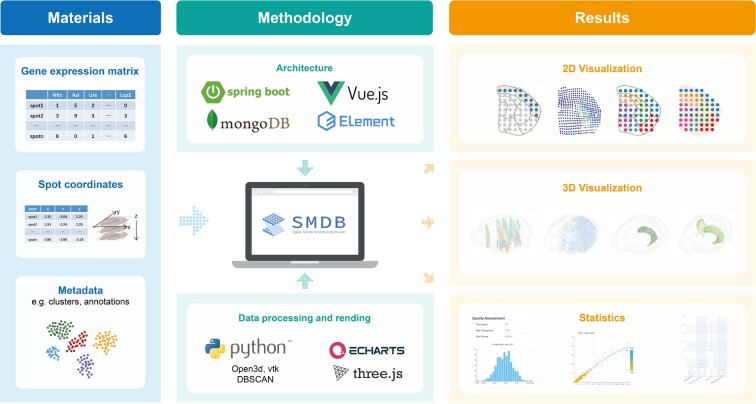

## INTRODUCTION

Spatially resolved transcriptomics has revolutionized our understanding of tissue organization by enabling *in situ* transcriptomic profiling, leading to groundbreaking discoveries in neuroscience (1–3), developmental biology (4) and disease research (5,6). ST data has been used to create unbiased spatial transcriptional landscapes of various tissues (7,8), including the brain (1–3,9), human kidney (10), heart (4), testis (11) and lung (12). Moreover, ST provides invaluable insights into the tissue disorganization observed in diseases, especially cancer, by revealing molecular features and mechanisms at the tumor microenvironment's leading edge with normal tissue. A comprehensive understanding of the biological functions reflected in the transcriptional state requires simultaneous knowledge about morphological context (13). Consequently, visualization tools that seamlessly combine spatial-omics and morphological information are becoming increasingly popular among researchers.

Several tools supporting spatial analysis and visualization have been released recently. Brain Explorer (14) allows the exploration of gene expression and anatomical information in 2D and 3D space using built-in reference data for adult mice. Other tools, such as spatialLIBD (15), Giotto (16), STUtility (17) and Cirrocumulus (https://github.com/lilab-bcb/cirrocumulus), enable users to load their data to analyze and visualize cell typing results with spatial positions in 2D space. Although ST-Viewer (https://github.com/jfnavarro/st_viewer) can present ST data in 3D space, it lacks support for morphology information. While these tools can visualize the gene expression pattern of adjacent 2D slices or align stacked experiments to create a static view of the tissue, it is inconvenient to screen the distribution of genes and related cell types from a 3D morphology perspective by traversing multiple slices simultaneously. There is an urgent need for a tool that can support the interactive exploration of molecular features and spatial morphology of ST data in 3D space.

The Spatial Multimodal Data Browser (SMDB) is a visualization tool designed to seamlessly connect gene expression profiles with spatial context and morphological information. It offers a range of features to enhance user experience and facilitate in-depth analysis, including:

- 2D spatial mapping that integrates molecular features from spatial transcriptomic data with morphological aspects like stained tissue sections.
- 3D morphological reconstruction and visualization based on gene expression, molecular subtypes or image segmentation. SMDB features a built-in reference atlas of Allen's mouse brain anatomy atlas for easy comparison with reconstructed morphology to investigate the differences.
- Interactive spot and region-level analysis, such as user-defined label filtering, and manual lassoing tools for outlining characteristic morphological regions with arbitrary shapes.
- Comprehensive summaries of ST data quality, as well as correlation and gene expression difference statistics between clusters.

## MATERIALS AND METHODS

### Implementation

SMDB is built with a Browser/Server architecture, leveraging Spring Boot for the backend and Vue and ElementUI frameworks for the frontend. The data is stored in MongoDB, a document-based NoSQL database. To process graphical data, SMDB employs open3d (18), DBSCAN (19) and vtk (https://vtk.org/). Echarts is used for loading 2D data, while Three.js library is utilized for rendering 3D geospatial data.

### Data workspace

SMDB is compatible with various spatial omics technologies and relies on three types of essential data: expression matrix, physical position of spots, and spot annotation information, along with optional anatomical structure data. Users must provide an expression matrix in TSV or TSV-based ZIP format, spot coordinates in a 4-column TSV file, and metadata-annotated spot information in TSV format. To illustrate its potential, SMDB utilized the Ortiz's dataset comprising 75 coronal sections of a hemisphere of the whole brain, alongside matching H&E-stained images and reference outlines (9). Additionally, SMDB allows users to load tissue outlines and offers convenient email notifications and workspace management for an enhanced user experience. Additional user data requirements and workspace usage can be found from SMDB website.

### 3D structure reconstruction

When dealing with ST datasets that include a reference anatomy atlas, the reconstruction process involves two crucial steps: 2D image registration and 3D reconstruction. Initially, each 2D section image is registered to a 3D stereotactic atlas such as the Allen CCFv3 (20), and the registered results serve as the foundation for 3D reconstruction. However, if the ST datasets lack a reference anatomy atlas, users can perform 3D reconstruction directly using molecular morphological features, spatial features of spots and other similar features.

The registration process for ST datasets usually involves three steps: locating the 2D slice position in the stereotactic atlas, re-slicing the reference slice image and registering the 2D slice to the reference slice image. There are currently three main types of registration methods: manual (21), semi-automatic (22), and automatic (23–25). The accuracy of these methods varies, with manual methods achieving up to 25 um accuracy, while semi-automated or automated methods typically range between 50 and 100 um. Although manual correction is often necessary to further improve registration accuracy, it cannot be fully automated, especially for studies requiring fine subregion segmentation of the brain. To aid users with registration, we have provided a detailed description of the registration process on the HELP page of SMDB. This allows users to perform registration locally and upload high-quality results to the browser for automatic 3D point cloud reconstruction.

The 3D reconstruction process contains three steps: noise erase, point cloud reconstruction, and smoothing. Before 3D reconstruction, we utilize the DBSCAN method (19) to eliminate noisy points through density-based clustering. This technique starts with a core point and continuously expands into areas of consistent density. Noise distributions, being more dispersed, cannot be classified into any clusters, and are consequently removed from the data. We then evaluate popular 3D reconstruction algorithms, including Convex Hull, Alpha Shape, Ball Pivoting, and Poisson Surface (refer to Supplementary Methods). Ultimately, we select the highly efficient Alpha Shape Algorithm (18) for point cloud reconstruction, as it effectively captures the external contours of the point cloud to facilitate 3D reconstruction. As the alpha shape algorithm forms triangular angles at the three intersecting points of the point cloud during contour formation, we further smooth the outer contours using standard low-pass filters in signal processing (26) in vtk. To evaluate the accuracy of our reconstruction, we utilized the Ortiz's dataset (9) to reconstruct different regions such as the hippocampal region and fiber tracts. Our 3D reconstructions exhibited high accuracy in matching the anatomical morphology, as detailed in the Supplementary Methods.

### Reference anatomy atlas collection

Given the widespread use of ST technology in neuroscience, SMDB has integrated the latest version of Allen Mouse Brain Common Coordinate Framework (CCFv3) (20), which includes 43 isocortical areas and their layers, 329 subcortical gray matter structures, 81 fiber tracts, and 8 ventricular structures. With SMDB, users can load reference anatomical structures into the workspace and accurately map different types of data into a common 3D space for comparison and correlation. This allows for a flexible and comprehensive approach to exploring tissue molecular landscapes and gaining insights into the complex interactions between gene expression and spatial context in the mouse brain.

## RESULTS

### 2D visualization

**Figure 1.**
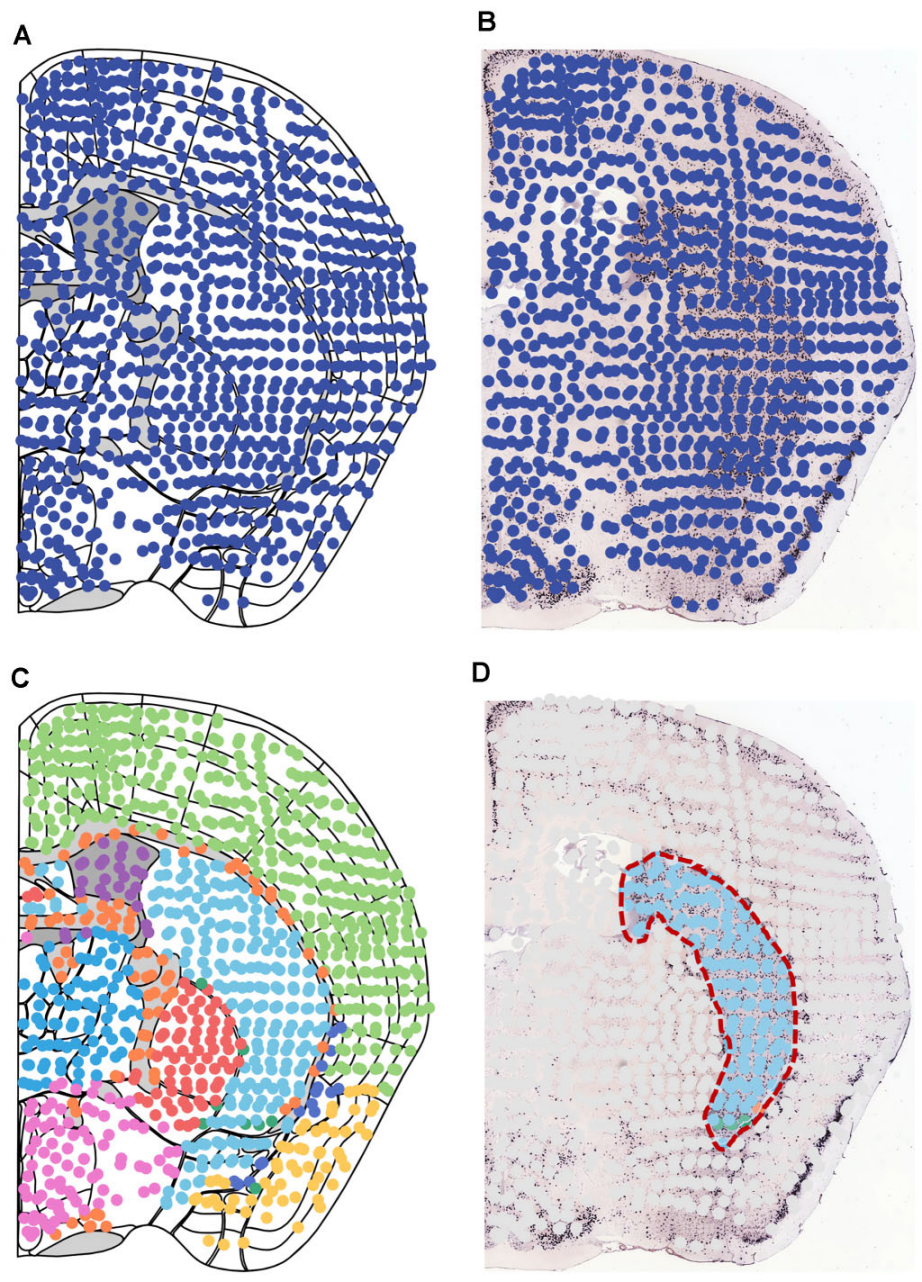
2D Visualization in SMDB. The ST data was obtained from Ortiz's molecular atlas of the adult mouse brain (–0.655 section). (**A**) Visualization of ST data aligned with Allen's mouse brain anatomy atlas borders; (**B**) Alignment and visualization of the same ST data based on *in situ* hybridization (ISH) image; (**C**) color-highlighted ST data corresponding to molecular clusters and aligned with Allen's mouse brain anatomy atlas borders; (**D**) a manually selected subregion highlighted based on background morphological information.

SMDB enables researchers to spatially integrate molecular data with morphological information in 2D space. Users can visualize spatially resolved transcriptomics data alongside references, such as Allen's mouse brain anatomy atlas borders (Figure 1A), or *in situ* hybridization (ISH) images to align molecular data with morphological regions (Figure 1B). SMDB highlights gene expression-profiled boundaries, revealing molecular characteristics within or between different regions. Employing supervised or unsupervised analysis clustering, researchers can segment regions or subregions to investigate tissue composition (Figure 1C).

Users can manually align morphology and spots by overlaying and scaling images freely. Using the lasso tool, 2D tissue sections can be segmented by interactively selecting regions of interest based on morphological information (Figure 1D). SMDB supports filtering spots in highlighted areas for subsequent annotation correlation, grouping statistics, leading edge analysis, and more.

### 3D visualization

**Figure 2.**
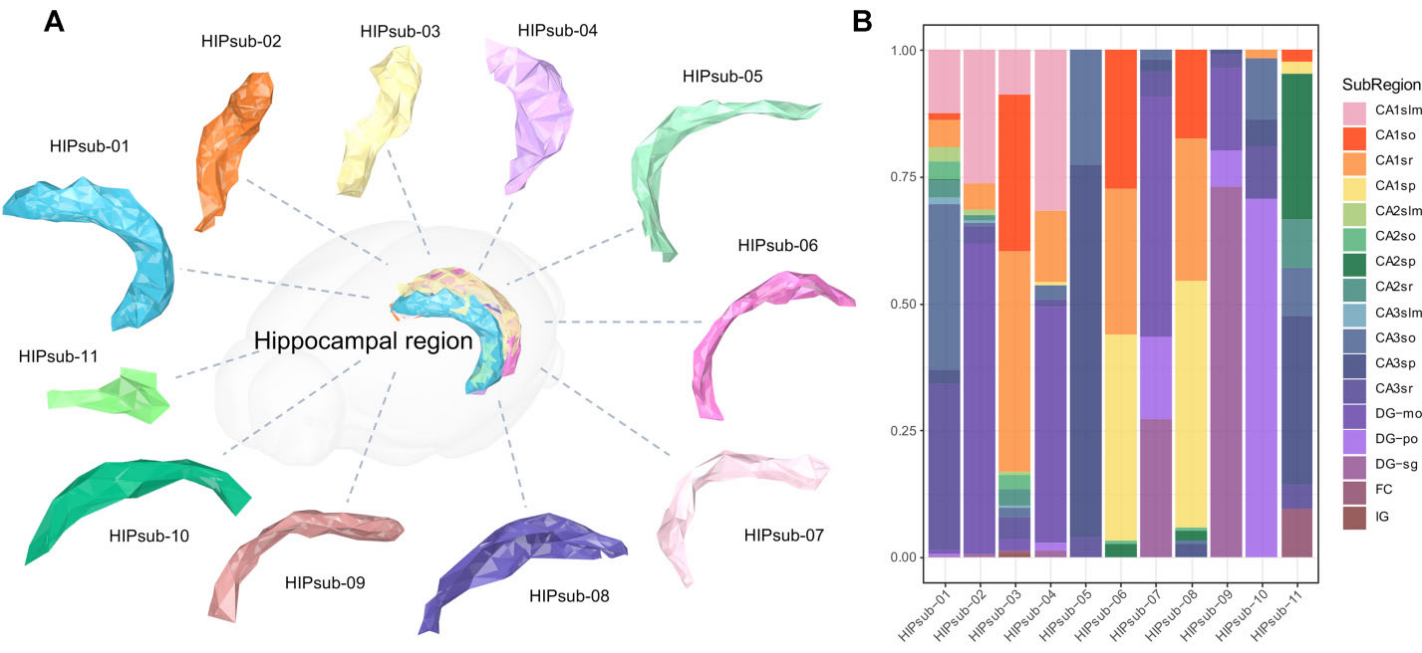
3D reconstruction of Hippocampal subregions based on molecular clusters. The ST data was derived from 11 molecular clusters within the hippocampal region of Ortiz's mouse brain molecular atlas. (**A**) Using SMDB’s 3D reconstruction module, a total of 11 sub-hippocampal regions (HIPsub) were identified and regenerated based on their molecular characteristics. (**B**) Barplot of the distribution of 11 HIPsub percentages across the major anatomical subregions of the hippocampal region. Each bar represents the ratio of spots within each HIPsub that were located in different anatomical regions, relative to the total number of spots in that HIPsub.

Various strategies for ST data visualization can applied in 3D space as a continuous superposition of information from multiple adjacent 2D slices in a third dimension (axis Z). SMDB enables the combined display of anatomical morphological features, molecular morphological features, spatial features of spots, and cell type features in the same workspace to interactively explore gene expression maps and the spatial distribution of cell types in tissues in 3D space (Figure 2).

The introduction of 3D spatial morphological reference contours allows for more accurate localization of the spatial position of different cell types and gene expression in tissues (Figure 2). By freely rotating the view and positioning molecular classification markers, SMDB enables users to reveal hidden patterns and molecular features that are easily missed in 2D perspective.

Unlike existing spatial transcriptome visualization tools that only align and stack 2D sections for display, SMDB includes a 3D reconstruction module that uses pure 3D tissue volume reconstruction technology to interactively display tissue morphology obtained by reconstructing tissue segmentation represented by anatomical features based on image slices, as well as molecular morphology obtained by features based on ST data. The reconstruction algorithm, optimized based on point cloud reconstruction, not only converts 3D coordinate point features into solid morphological features but also preserves morphological details with fine tuning (Figure 2A). Certain morphological features were concentrated in distinct anatomical regions (Figure 2B); for example, HIPsub-10 was prevalent in DG-po, HIPsub-09 in DG-sp, and HIPsub-05 in CA1sp. Some HIPsubs further divided anatomical structures into molecular clusters, such as Hipsub-02,04,07 which constituted DG-mo. Others encompassed multiple regions like HIPsub-01, while some necessitated further exploration as they did not clearly correspond to a specific region. This method connects the physical structure of biological systems to molecular characteristics, providing researchers with a comprehensive understanding of tissue molecular landscapes.

## CASE STUDY

The dorsal striatum (DS) in rodents is a single mass of gray matter often referred to as the caudate-putamen complex (27), and comprises two functionally distinct regions: the dorsolateral striatum (DLS), responsible for integrating sensorimotor information, and the dorsomedial striatum (DMS), involved in processing associative information (28–31). However, the morphological boundary of the DLS and DMS remains to be clearly defined.

**Figure 3.**
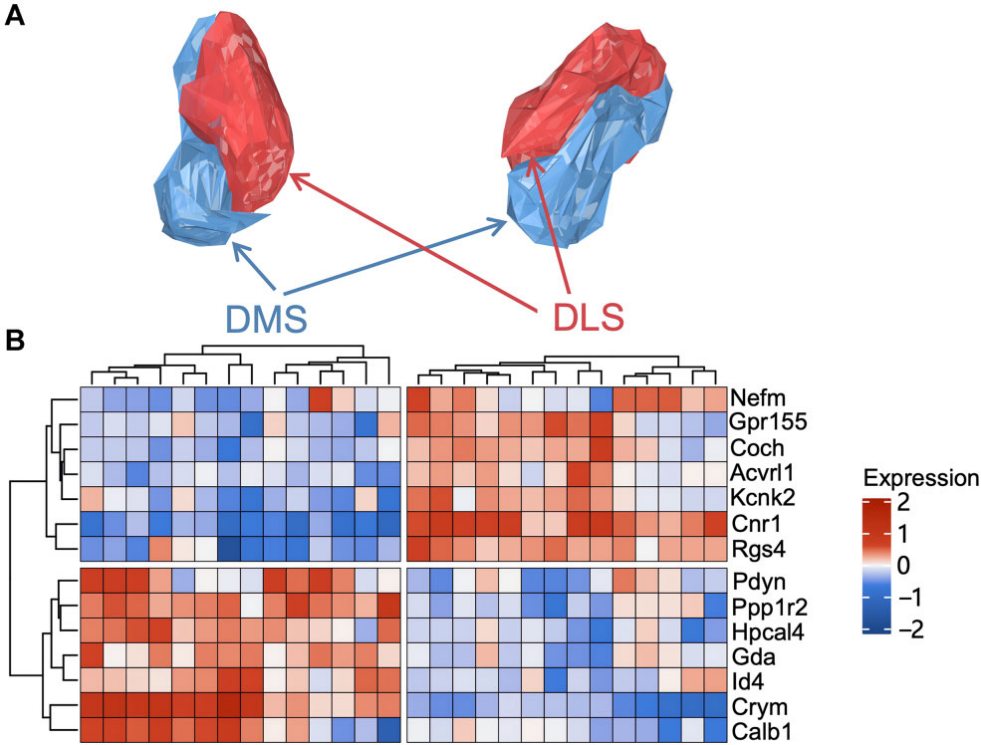
The morphological clues of the DLS and DMS subregions. (**A**) 3D reconstructions of morphological boundary of the DLS and DMS. (**B**) Heatmap of the differential expression genes (DEGs) of DLS and DMS reconstructed morphological regions.

Using the SMDB, we identified two primary subregions of the DS in the striatum, based on molecular subtypes from the Ortiz's mouse brain molecular atlas (9). We analyzed the differential expression genes (DEGs) of these two subregions and discovered Crym and Cnr1 as typical markers for DMS and DLS, corresponding to specific markers of two distinct medium spiny neuron (MSN) subtypes (32). We then delineate the morphological subregions of these two genes, overlaying Allen's mouse brain anatomy atlas borders on each slice. Subsequently, we utilized the 3D reconstruction tool in SMDB to create separate 3D representations of the DMS and DLS regions (Figure 3A).

We extracted the spots of each region in SMDB and explored the molecular differences between the DLS and DMS morphological regions. We identified 14 ISH-supported regional signature genes (Figure 3B, Supplementary Table 1), including Coch, Kcnk2 and Calb1, etc. which have been previously reported to be associated with DLS and DMS regions (33–37). SMDB offers insights into the morphological characterization of the DLS and DMS subregions through a combination of molecular and morphological approaches.

### Comparison with other tools

Several tools are available for visualizing ST data combined with 2D morphological information. Table 1 compares SMDB with several well-known tools supporting ST data visualization, including Giotto (16), SpatialLIBD (15), STUtility (17), ST Viewer and Cirrocumulus (Table1, Supplementary Table 2). While most of these offline tools cover 2D spatial visualization functions (raw slices form of ST data), SMDB stands out as the only visualization tool that can cover embedded 3D structure, customized outline, clustering selection, and 3D reconstruction. What's more, SMDB is web-based and runs directly in the browser, eliminating the need for a local installation process. Its compatibility with different ST technologies and incorporation of the Allen Mouse Brain Common Coordinate Framework (CCFv3) further expands its application in neuroscience research.

**Table 1.** A comparison of SMDB and other visualization tools

	**SMDB**	**Giotto viewer**	**SpatialLIBD**	**STUtility**	**ST viewer**	**Cirrocumulus**
**Online service**	Yes	-	-	-	-	-
**2D visualization features**						
**Images overlay**	Yes	Yes	Yes	Yes	Yes	Yes
**Manual annotation**	Yes	Yes	Yes	Yes	-	-
**Feature plot**	Yes	Yes	Yes	Yes	Yes	Yes
**Clustering selection**	Yes	Yes	Yes	-	Yes	Yes
**3D visualization features**						
**Embedded 3D structure**	Yes	-	-	-	Yes	-
**Customizing outline**	Yes	-	-	-	-	-
**Clustering selection**	Yes	-	-	-	-	-
**Feature plot**	Yes	-	-	Yes	Yes	-
**3D reconstruction**	Yes	-	-	-	-	-

## DISCUSSION

SMDB is a versatile tool that empowers researchers to interpret cell composition and gene expression in tissues in both 2D and 3D space. Users can visualize molecular expression information with reference atlases, morphological segmented sections without a reference atlas, or customized partition information using a lasso tool for challenging annotations.

In the future, SMDB aims to expand its capabilities as an online visualization web service by enhancing its functionality to include more sophisticated analyses based on spatial information, such as cell type annotation, spatial domain identification, and cell–cell communication. By integrating high-throughput proteomics approaches with spatial transcriptomics, SMDB plans to improve compatibility with both transcriptome and protein expression, further strengthening the joint analysis and visualization of multi-omics data. Additionally, the tool will continue to incorporate research on reference spatial structures for various tissues and organs, leveraging reconstructed 3D tissue morphology based on stained images and molecular expression information to facilitate a deeper understanding of tissue structure and function for researchers.

In summary, SMDB is an innovative and powerful tool that enables the exploration of molecular landscapes in tissues across 2D and 3D spaces. Its adaptability and applicability in multiple spatial omics fields make it an invaluable resource for researchers.

## DATA AVAILABILITY

SMDB is available at https://www.biosino.org/smdb. The application is free and open to all users with no login requirement.

## Supplementary Material

gkad413_Supplemental_Files

## References

[1] BRAIN Initiative Cell Census Network A multimodal cell census and atlas of the mammalian primary motor cortex. Nature. 2021; 598:86–102. 34616075 10.1038/s41586-021-03950-0 PMC8494634

[2] Moffitt J.R. , Bambah-Mukku D., Eichhorn S.W., Vaughn E., Shekhar K., Perez J.D., Rubinstein N.D., Hao J., Regev A., Dulac C. et al. Molecular, spatial, and functional single-cell profiling of the hypothalamic preoptic region. Science. 2018; 362:eaau5324. 30385464 10.1126/science.aau5324 PMC6482113

[3] Gyllborg D. , Langseth C.M., Qian X., Choi E., Salas S.M., Hilscher M.M., Lein E.S., Nilsson M. Hybridization-based in situ sequencing (HybISS) for spatially resolved transcriptomics in human and mouse brain tissue. Nucleic Acids Res. 2020; 48:e112. 32990747 10.1093/nar/gkaa792 PMC7641728

[4] Asp M. , Giacomello S., Larsson L., Wu C., Furth D., Qian X., Wardell E., Custodio J., Reimegard J., Salmen F. et al. A spatiotemporal organ-wide gene expression and cell atlas of the developing human heart. Cell. 2019; 179:1647–1660. 31835037 10.1016/j.cell.2019.11.025

[5] Thrane K. , Eriksson H., Maaskola J., Hansson J., Lundeberg J. Spatially resolved transcriptomics enables dissection of genetic heterogeneity in stage III cutaneous malignant melanoma. Cancer Res. 2018; 78:5970–5979. 30154148 10.1158/0008-5472.CAN-18-0747

[6] Chen W.T. , Lu A., Craessaerts K., Pavie B., Sala Frigerio C., Corthout N., Qian X., Lalakova J., Kuhnemund M., Voytyuk I. et al. Spatial transcriptomics and in situ sequencing to study Alzheimer's disease. Cell. 2020; 182:976–991. 32702314 10.1016/j.cell.2020.06.038

[7] Rao A. , Barkley D., Franca G.S., Yanai I. Exploring tissue architecture using spatial transcriptomics. Nature. 2021; 596:211–220. 34381231 10.1038/s41586-021-03634-9 PMC8475179

[8] Tian L. , Chen F., Macosko E.Z. The expanding vistas of spatial transcriptomics. Nat. Biotechnol. 2022; 10.1038/s41587-022-01448-2. PMC10091579 36192637

[9] Ortiz C. , Navarro J.F., Jurek A., Martin A., Lundeberg J., Meletis K. Molecular atlas of the adult mouse brain. Sci Adv. 2020; 6:eabb3446. 32637622 10.1126/sciadv.abb3446 PMC7319762

[10] Lake B.B. , Menon R., Winfree S., Hu Q., Ferreira R.M., Kalhor K., Barwinska D., Otto E.A., Ferkowicz M., Diep D. et al. An atlas of healthy and injured cell states and niches in the human kidney. 2021; bioRxiv doi:29 July 2021, preprint: not peer reviewed 10.1101/2021.07.28.454201. PMC10356613 37468583

[11] Chen H. , Murray E., Sinha A., Laumas A., Li J., Lesman D., Nie X., Hotaling J., Guo J., Cairns B.R. et al. Dissecting mammalian spermatogenesis using spatial transcriptomics. Cell Rep. 2021; 37:109915. 34731600 10.1016/j.celrep.2021.109915 PMC8606188

[12] Madissoon E. , Oliver A.J., Kleshchevnikov V., Wilbrey-Clark A., Polanski K., Orsi A.R., Mamanova L., Bolt L., Richoz N., Elmentaite R. et al. A spatial multi-omics atlas of the human lung reveals a novel immune cell survival niche. 2021; bioRxiv doi:27 November 2021, preprint: not peer reviewed 10.1101/2021.11.26.470108.

[13] Stahl P.L. , Salmen F., Vickovic S., Lundmark A., Navarro J.F., Magnusson J., Giacomello S., Asp M., Westholm J.O., Huss M. et al. Visualization and analysis of gene expression in tissue sections by spatial transcriptomics. Science. 2016; 353:78–82. 27365449 10.1126/science.aaf2403

[14] Lau C. , Ng L., Thompson C., Pathak S., Kuan L., Jones A., Hawrylycz M. Exploration and visualization of gene expression with neuroanatomy in the adult mouse brain. BMC Bioinformatics. 2008; 9:153. 18366675 10.1186/1471-2105-9-153 PMC2375125

[15] Pardo B. , Spangler A., Weber L.M., Page S.C., Hicks S.C., Jaffe A.E., Martinowich K., Maynard K.R., Collado-Torres L. spatialLIBD: an R/Bioconductor package to visualize spatially-resolved transcriptomics data. BMC Genomics. 2022; 23:434. 35689177 10.1186/s12864-022-08601-w PMC9188087

[16] Dries R. , Zhu Q., Dong R., Eng C.L., Li H., Liu K., Fu Y., Zhao T., Sarkar A., Bao F. et al. Giotto: a toolbox for integrative analysis and visualization of spatial expression data. Genome Biol. 2021; 22:78. 33685491 10.1186/s13059-021-02286-2 PMC7938609

[17] Bergenstrahle J. , Larsson L., Lundeberg J. Seamless integration of image and molecular analysis for spatial transcriptomics workflows. BMC Genomics. 2020; 21:482. 32664861 10.1186/s12864-020-06832-3 PMC7386244

[18] Teichmann M. , Capps M. Proceedings Visualization '98 (Cat. No.98CB36276). 1998; 67–72.

[19] Rui Y.C. , Zhou Z.L., Cai X., Dong L.J. A novel robust method for acoustic emission source location using DBSCAN principle. Measurement. 2022; 191:110812–110812.

[20] Wang Q. , Ding S.L., Li Y., Royall J., Feng D., Lesnar P., Graddis N., Naeemi M., Facer B., Ho A. et al. The Allen mouse brain common coordinate framework: a 3D reference atlas. Cell. 2020; 181:936–953. 32386544 10.1016/j.cell.2020.04.007 PMC8152789

[21] Yates S.C. , Groeneboom N.E., Coello C., Lichtenthaler S.F., Kuhn P.H., Demuth H.U., Hartlage-Rubsamen M., Rossner S., Leergaard T., Kreshuk A. et al. QUINT: workflow for quantification and spatial analysis of features in histological images from rodent brain. Front. Neuroinform. 2019; 13:75. 31849633 10.3389/fninf.2019.00075 PMC6901597

[22] Xiong J. , Ren J., Luo L., Horowitz M. Mapping histological slice sequences to the Allen mouse brain atlas without 3D reconstruction. Front Neuroinform. 2018; 12:93. 30618698 10.3389/fninf.2018.00093 PMC6297281

[23] Carey H. , Pegios M., Martin L., Saleeba C., Turner A., Everett N., Puchades M., Bjaalie J., McMullan S. DeepSlice: rapid fully automatic registration of mouse brain imaging to a volumetric atlas. 2022; bioRxiv doi:30 April 2022, preprint: not peer reviewed 10.1101/2022.04.28.489953. PMC10514056 37735467

[24] Xu J. , Moyer D., Grant P.E., Golland P., Iglesias J.E., Adalsteinsson E. SVoRT: Iterative Transformer for Slice-to-Volume Registration in Fetal Brain MRI. 2022; Nature Switzerland, Cham Springer 3–13. 10.1007/978-3-031-16446-0_1 PMC10129054 37103480

[25] Furth D. , Vaissiere T., Tzortzi O., Xuan Y., Martin A., Lazaridis I., Spigolon G., Fisone G., Tomer R., Deisseroth K. et al. An interactive framework for whole-brain maps at cellular resolution. Nat. Neurosci. 2018; 21:139–149. 29203898 10.1038/s41593-017-0027-7 PMC5994773

[26] Taubin G. , Zhang T., Golub G. Optimal surface smoothing as filter design. 1996; Berlin, Heidelberg Springer Berlin Heidelberg 283–292.

[27] Yarom O. , Cohen D Putative cholinergic interneurons in the ventral and dorsal regions of the striatum have distinct roles in a two choice alternative association task. Front. Syst. Neurosci. 2011; 5:36. 21660109 10.3389/fnsys.2011.00036 PMC3106210

[28] Joel D. , Weiner I. The organization of the basal ganglia-thalamocortical circuits: open interconnected rather than closed segregated. Neuroscience. 1994; 63:363–379. 7891852 10.1016/0306-4522(94)90536-3

[29] Yin H.H. , Knowlton B.J., Balleine B.W. Inactivation of dorsolateral striatum enhances sensitivity to changes in the action-outcome contingency in instrumental conditioning. Behav. Brain Res. 2006; 166:189–196. 16153716 10.1016/j.bbr.2005.07.012

[30] Graybiel A.M. Habits, rituals, and the evaluative brain. Annu. Rev. Neurosci. 2008; 31:359–387. 18558860 10.1146/annurev.neuro.29.051605.112851

[31] Humphries M.D. , Prescott T.J. The ventral basal ganglia, a selection mechanism at the crossroads of space, strategy, and reward. Prog. Neurobiol. 2010; 90:385–417. 19941931 10.1016/j.pneurobio.2009.11.003

[32] Gokce O. , Stanley G.M., Treutlein B., Neff N.F., Camp J.G., Malenka R.C., Rothwell P.E., Fuccillo M.V., Sudhof T.C., Quake S.R. Cellular taxonomy of the mouse striatum as revealed by single-cell RNA-Seq. Cell Rep. 2016; 16:1126–1137. 27425622 10.1016/j.celrep.2016.06.059 PMC5004635

[33] Martin A. , Calvigioni D., Tzortzi O., Fuzik J., Warnberg E., Meletis K. A spatiomolecular map of the striatum. Cell Rep. 2019; 29:4320–4333. 31875543 10.1016/j.celrep.2019.11.096

[34] Djillani A. , Mazella J., Heurteaux C., Borsotto M. Role of TREK-1 in health and disease, focus on the central nervous system. Front. Pharmacol. 2019; 10:379. 31031627 10.3389/fphar.2019.00379 PMC6470294

[35] Pan J. , Yu J., Sun L., Xie C., Chang L., Wu J., Hawes S., Saez-Atienzar S., Zheng W., Kung J. et al. ALDH1A1 regulates postsynaptic mu-opioid receptor expression in dorsal striatal projection neurons and mitigates dyskinesia through transsynaptic retinoic acid signaling. Sci. Rep. 2019; 9:3602. 30837649 10.1038/s41598-019-40326-x PMC6401150

[36] Brimblecombe K.R. , Cragg S.J. The striosome and matrix compartments of the striatum: a path through the labyrinth from neurochemistry toward Function. ACS Chem Neurosci. 2017; 8:235–242. 27977131 10.1021/acschemneuro.6b00333

[37] Stanley G. , Gokce O., Malenka R.C., Sudhof T.C., Quake S.R. Continuous and discrete neuron types of the adult murine striatum. Neuron. 2020; 105:688–699. 31813651 10.1016/j.neuron.2019.11.004

