# Supplementary material for: SMDB: a Spatial Multimodal Data Browser
Source: Nucleic Acids Res. 2023 May 22;51(W1):W553–9. doi: 10.1093/nar/gkad413 (PMC10320082; doi:10.1093/nar/gkad413)
Supplement: gkad413_Supplemental_Files [file gkad413_supplemental_files.zip › Supplementary_Methods_20230502-1.docx]

# Supplementary Methods for 3D reconstruction processes

## Noise erase

Noisy data will make the reconstructed shape different from the ground truth and affect the final reconstruction result (Fig. SM1). Therefore, before reconstruction, we first use the data clustering method to erase the noisy data. For the spots in spatial transcriptomics (ST) data that needs to be reconstructed in a specific region, the point cloud data in the core region is dense and there are some isolated spots. The most appropriate method is density-based clustering and the most common method in density-based clustering is DBSCAN.


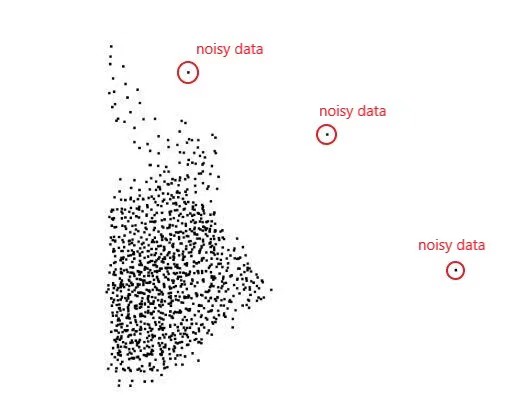


Figure SM1. Description of the noisy data.

The DBSCAN algorithm has three types of points (core points, boundary points and noise points) and two types of parameters (eps, E neighborhood) and minPts, minimum points) (1). In the first step, DBSCAN randomly selects an unvisited point to find all neighborhood points. If the number of neighborhood points in the ring with the selected point as the center and the radius of eps is greater than minPts, the selected point and its neighborhood points form a cluster. The selected point is called the core point, set as the access point, and the neighborhood point is called the boundary point. A cluster consists of boundary points. Parameter minPts identifies the clustering that requires at least minPts points, and parameter eps identifies the neighborhood points within the clustering radius centered on the core point. In the second step, repeat the process for each unassessed point in the cluster to expand the cluster. However, if the number of neighborhood points within the ring is less than minPts, the selected points will be set to noise points. The DBSCAN algorithm repeats steps 1 and 2 to obtain new clusters and noise points. For ST data, the 3D cloud point is collected by a continuous superposition of adjacent 2D slices in a third dimension (axis Z), so it can be divided into N sections. And then, the DBSCAN algorithm erases the noisy data for each section.


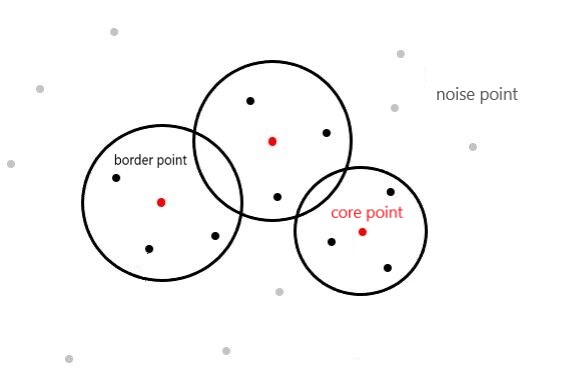


Figure SM2. the principle of the DBSCAN algorithm.

## 3D reconstruction

After noise erasing, the point cloud reconstruction algorithm can reconstruct the points in the region into 3D polygons. There are many point cloud 3D reconstruction algorithms, such as Ball Pivoting (2), Convex Hull (3), Poisson Surface (4), Alpha Shape (5), etc. The reconstruction results of four different algorithms are shown in Figure SM3.


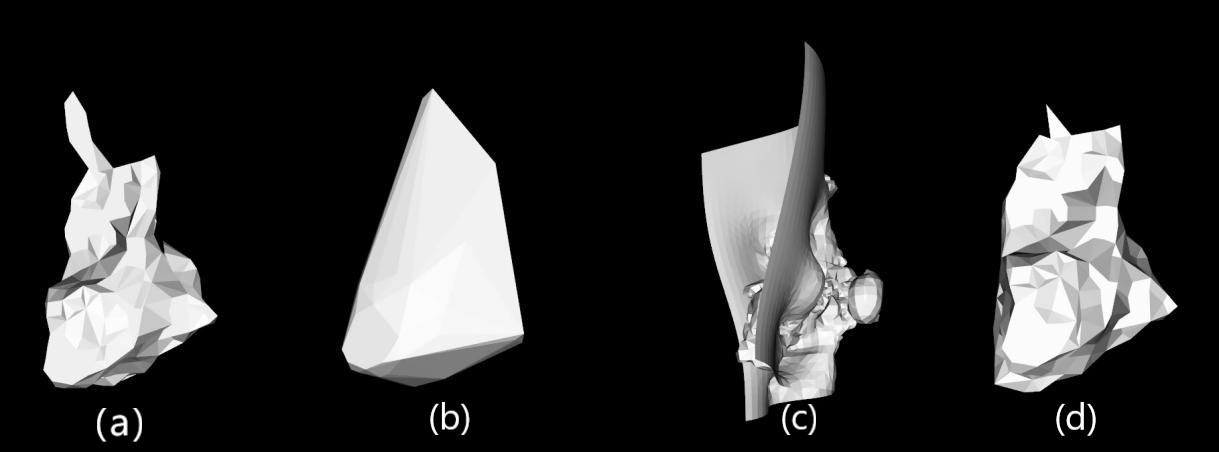


Figure SM3. The reconstruction results of the four algorithms. a). Ball Pivoting Algorithm (radii=0.5), b). Convex Hull Algorithm, c). Poisson Surface Algorithm (depth=9) (d) Alpha Shape Algorithm (alpha=0.5).

As the distribution of the point cloud is not hollow, the Poisson Surface is the least well reconstructed. The Convex Hull algorithm only produces a convex hull with a smooth surface, which loses concave and convex information. Although the results of Ball Pivoting and Alpha Shape are similar, Ball Pivoting requires a point cloud with vertex normals, whereas Alpha Shape does not. Therefore, we choose the Alpha Shape as our 3D reconstruction algorithm.

Alpha Shape algorithm is a point cloud reconstruction algorithm for 3D model reconstruction. The basic principle of the algorithm is to roll the ball on a point cloud. During rolling, the three points that intersect the ball form a triangle. The radius of the ball determines the quality of the reconstruction. If the radius is large, the result is close to the Convex Hull algorithm. Instead, the reconstructed shape is close to the point cloud. This method can obtain polygon data of point cloud contour.

## Smooth process

To bring the reconstruction results closer to the anatomical morphology, the surface of the 3D model has to be smoothed. The comparison results before and after smoothing are shown in Figure SM4.


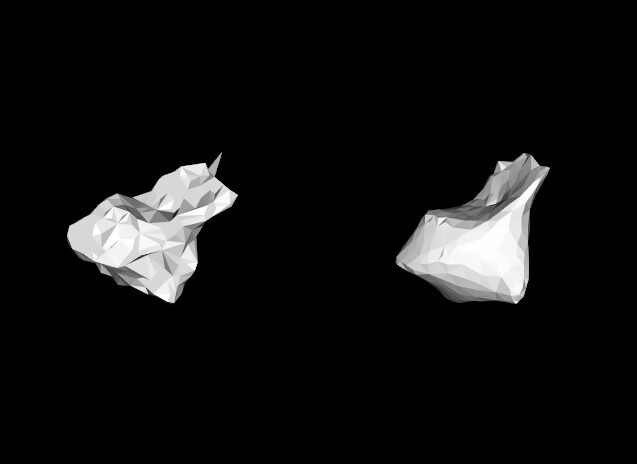


Figure SM4. The comparison results before and after smoothing. The smooth iteration is set to 15, and passband is set to 0.1.

Standard signal processing low-pass filters (6) is used to smooth the reconstructed result. The windowed function *sinc* is used for poly data smoothing, which is similar to Laplace smoothing. Through continuous iteration, relatively smooth poly data is obtained. Unlike Laplace smoothing, this method can avoid shrinking the reconstruction result. The smooth algorithm requires two parameters. The first parameter *smooth iteration* means the value of smoothing times. The second parameter is *passband* for the windowed *sinc* filter. The Value of *passband* is between 0 and 2. The more values of smooth iteration and passband produce more smoothing.

## Evaluation

We chose the algorithms for 3D reconstruction and evaluated their effectiveness using three metrics: Root Mean Squared Error (RMSE), Average Euclidean distance (AED), and Standard deviation Euclidean distance (SDED). Our ground truth consisted of 13 main regions from the Allen Mouse Brain Common Coordinate Framework (CCFv3) (7), with the 3D reconstruction results serving as predicted values.

RMSE measures the average squared difference between the predicted and ground truth values, and can be calculated using the following formula:

$$RMSE=\sqrt{\frac{\sum_{i=1}^{N} \left( S_{i}-S_{i}^{'} \right)^{2}}{n}}$$

Here, $S_{i}$ represents the predicted value, $S_{i}^{'}$ represents the ground truth value, and n is the total number of point clouds.

The Euclidean Distance is a straight-line distance measurement between two points in an n-dimensional space. For our evaluation, we utilized Average Euclidean Distance (AED) and Standard deviation Euclidean Distance (SDED) as additional metrics. AED represents the average distance, while SDED represents the sample standard deviation of the distances.

The equations for AED and SDED are as follows:

$$AED=\frac{2}{n\left( n-1 \right)}\sum_{i=1}^{n-1} \sum_{j=i+1}^{n} d_{ij}$$

$$SDED=\sqrt{\frac{\sum_{i=1}^{n-1} \sum_{j=i+1}^{n} \left( d_{ij}-\mathrm{AED} \right)^{2}}{n\left( n-1 \right)}}$$

Here, n represents the number of data points, while $d_{ij}$ denotes the Euclidean distance between points i and j.

### Loss distance analysis

We evaluated four 3D reconstruction algorithms (Ball Pivoting, Convex Hull, Poisson Surface ,and Alpha Shape algorithms) using datasets from different brain regions in Ortiz's dataset (8). Table 1 displays the results, indicating that the Alpha Shape algorithm outperforms the others on most datasets. Additionally, Alpha Shape and Ball Pivoting have similar performance on a small portion of the dataset due to their similar reconstruction principles. We also computed the average value of each evaluation metric across all datasets, presented in Table SM2, which further confirms that Alpha Shape produces the best results.

Table SM1. Quantitative 3D Reconstruction Results. This table compares the performance of various 3D reconstruction algorithms across different datasets, with the top-performing outcomes highlighted in bold.

| Dataset | Algorithm | RMSE | ED  (Average) | ED  (Standard deviation) |
| --- | --- | --- | --- | --- |
| Cerebellum | Ball Pivoting | **0.118** | **0.106** | **0.051** |
|  | Convex Hull | 0.123 | 0.111 | 0.053 |
|  | Poisson Surface | 0.359 | 0.292 | 0.208 |
|  | Alpha Shape | 0.122 | 0.111 | **0.051** |
| Cortical subplate | Ball Pivoting | 0.291 | 0.240 | 0.164 |
|  | Convex Hull | **0.257** | 0.226 | **0.123** |
|  | Poisson Surface | 0.367 | 0.280 | 0.238 |
|  | Alpha Shape | 0.260 | **0.209** | **0.155** |
| Hindbrain | Ball Pivoting | 0.359 | 0.274 | 0.231 |
|  | Convex Hull | 0.514 | 0.252 | 0.449 |
|  | Poisson Surface | 0.936 | 0.602 | 0.781 |
|  | Alpha Shape | **0.296** | **0.221** | **0.197** |
| Hippocampal region | Ball Pivoting | 0.258 | 0.216 | 0.140 |
|  | Convex Hull | 0.121 | 0.107 | 0.057 |
|  | Poisson Surface | 0.614 | 0.393 | 0.472 |
|  | Alpha Shape | **0.113** | **0.100** | **0.054** |
| Hypothalamus | Ball Pivoting | 0.270 | 0.225 | 0.158 |
|  | Convex Hull | **0.238** | 0.199 | **0.148** |
|  | Poisson Surface | 0.458 | 0.330 | 0.326 |
|  | Alpha Shape | **0.238** | **0.193** | 0.157 |
| Isocortex | Ball Pivoting | 0.339 | 0.288 | **0.193** |
|  | Convex Hull | 0.301 | 0.188 | 0.245 |
|  | Poisson Surface | 0.467 | 0.332 | 0.335 |
|  | Alpha Shape | **0.269** | **0.186** | 0.224 |
| Midbrain | Ball Pivoting | 0.395 | 0.324 | 0.232 |
|  | Convex Hull | 0.476 | 0.349 | 0.327 |
|  | Poisson Surface | 0.638 | 0.462 | 0.442 |
|  | Alpha Shape | **0.322** | **0.236** | **0.224** |
| Olfactory areas | Ball Pivoting | **0.278** | **0.220** | **0.176** |
|  | Convex Hull | 0.389 | 0.277 | 0.291 |
|  | Poisson Surface | 0.859 | 0.548 | 0.665 |
|  | Alpha Shape | 0.327 | 0.245 | 0.226 |
| Pallidum | Ball Pivoting | **0.746** | **0.416** | **0.619** |
|  | Convex Hull | 1.070 | 0.701 | 0.810 |
|  | Poisson Surface | 1.217 | 0.900 | 0.822 |
|  | Alpha Shape | 0.767 | 0.431 | 0.635 |
| Retrohippocampal | Ball Pivoting | 0.222 | 0.182 | **0.127** |
|  | Convex Hull | 0.230 | 0.181 | 0.141 |
|  | Poisson Surface | 0.719 | 0.455 | 0.556 |
|  | Alpha Shape | **0.207** | **0.159** | 0.132 |
| Striatum | Ball Pivoting | 0.267 | 0.218 | 0.153 |
|  | Convex Hull | 0.213 | 0.170 | 0.129 |
|  | Poisson Surface | 0.410 | 0.297 | 0.283 |
|  | Alpha Shape | **0.174** | **0.142** | **0.101** |
| Fiber tracts | Ball Pivoting | 0.134 | 0.115 | 0.069 |
|  | Convex Hull | 0.160 | 0.131 | 0.094 |
|  | Poisson Surface | 0.565 | 0.327 | 0.477 |
|  | Alpha Shape | **0.127** | **0.108** | **0.066** |
| Ventricular | Ball Pivoting | 0.119 | 0.103 | 0.059 |
|  | Convex Hull | **0.076** | **0.069** | **0.033** |
|  | Poisson Surface | 0.531 | 0.437 | 0.434 |
|  | Alpha Shape | 0.114 | 0.100 | 0.054 |

Table SM2. Averaged Performance Metrics from Table 1. This table presents the average results calculated from Table 1, with the best-performing values emphasized in bold.

| Algorithm | RMSE | AED | SDED |
| --- | --- | --- | --- |
| Ball Pivoting | 0.292 | 0.225 | 0.182 |
| Convex Hull | 0.321 | 0.228 | 0.223 |
| Poisson Surface | 0.626 | 0.435 | 0.464 |
| Alpha Shape | **0.257** | **0.188** | **0.175** |

### Alpha parameter assessments

We examined the impact of different alpha values in the Alpha Shape algorithm across various datasets, setting alpha values to 0.1, 0.25, 0.5, 0.75, 1, and 1.5. Table 3 displays the quantitative results of this analysis. Notably, when alpha was set to 0.1, some datasets failed to reconstruct, which is also depicted in Fig. SM5. Additionally, when alpha exceeded 0.5, the performance of the reconstruction results remained consistent.

Table SM3. Alpha Value Quantitative Analysis. This table compares the performance of different alpha values for the Alpha Shape algorithm across various datasets, with "NaN" indicating no result.

| Dataset | α | RMSE | AED | SDED | Reconstruction  evaluation |
| --- | --- | --- | --- | --- | --- |
| Cerebellum | 0.1 | NaN | NaN | NaN | failed |
|  | 0.25 | 0.134 | 0.125 | 0.049 | with holes |
|  | 0.5 | 0.122 | 0.111 | 0.051 | normal |
|  | 0.75 | 0.117 | 0.106 | 0.049 | normal |
|  | **1** | **0.115** | **0.104** | **0.049** | **normal** |
|  | 1.5 | 0.115 | 0.105 | 0.048 | normal |
| Cortical subplate | 0.1 | NaN | NaN | NaN | failed |
|  | 0.25 | 0.260 | 0.209 | 0.154 | with holes |
|  | 0.5 | 0.260 | 0.209 | 0.155 | normal |
|  | **0.75** | **0.255** | **0.207** | **0.149** | **normal** |
|  | 1 | 0.263 | 0.214 | 0.153 | normal |
|  | 1.5 | 0.253 | 0.212 | 0.138 | normal |
| Hindbrain | 0.1 | 0.278 | 0.244 | 0.136 | failed |
|  | 0.25 | 0.289 | 0.228 | 0.178 | with holes |
|  | **0.5** | **0.296** | **0.221** | **0.197** | **normal** |
|  | 0.75 | 0.316 | 0.237 | 0.210 | normal |
|  | 1 | 0.341 | 0.253 | 0.230 | normal |
|  | 1.5 | 0.376 | 0.270 | 0.263 | normal |
| Hippocampal region | 0.1 | 0.195 | 0.179 | 0.076 | failed |
|  | 0.25 | 0.135 | 0.117 | 0.068 | with holes |
|  | 0.5 | 0.113 | 0.100 | 0.054 | normal |
|  | 0.75 | 0.108 | 0.096 | 0.050 | normal |
|  | **1** | **0.107** | **0.095** | **0.051** | **normal** |
|  | 1.5 | 0.109 | 0.097 | 0.051 | normal |
| Hypothalamus | 0.1 | 0.243 | 0.212 | 0.118 | failed |
|  | 0.25 | 0.239 | 0.193 | 0.157 | with holes |
|  | **0.5** | **0.238** | **0.193** | **0.157** | **normal** |
|  | 0.75 | 0.244 | 0.200 | 0.159 | normal |
|  | 1 | 0.251 | 0.208 | 0.163 | normal |
|  | 1.5 | 0.251 | 0.211 | 0.161 | normal |
| Isocortex | 0.1 | 0.341 | 0.307 | 0.147 | failed |
|  | 0.25 | 0.292 | 0.225 | 0.208 | with holes |
|  | **0.5** | **0.269** | **0.186** | **0.224** | **normal** |
|  | 0.75 | 0.279 | 0.190 | 0.235 | normal |
|  | 1 | 0.272 | 0.189 | 0.229 | normal |
|  | 1.5 | 0.244 | 0.175 | 0.203 | normal |
| Midbrain | 0.1 | 0.197 | 0.180 | 0.081 | failed |
|  | 0.25 | 0.314 | 0.242 | 0.206 | with holes |
|  | **0.5** | **0.322** | **0.236** | **0.224** | **normal** |
|  | 0.75 | 0.347 | 0.254 | 0.243 | normal |
|  | 1 | 0.355 | 0.263 | 0.245 | normal |
|  | 1.5 | 0.375 | 0.280 | 0.257 | normal |
| Olfactory areas | 0.1 | 0.238 | 0.219 | 0.091 | failed |
|  | 0.25 | 0.278 | 0.219 | 0.177 | with holes |
|  | **0.5** | **0.327** | **0.245** | **0.226** | **normal** |
|  | 0.75 | 0.339 | 0.250 | 0.240 | normal |
|  | 1 | 0.346 | 0.255 | 0.245 | normal |
|  | 1.5 | 0.346 | 0.255 | 0.248 | normal |
| Pallidum | 0.1 | NaN | NaN | NaN | failed |
|  | 0.25 | 0.752 | 0.421 | 0.623 | with holes |
|  | **0.5** | **0.767** | **0.431** | **0.635** | **normal** |
|  | 0.75 | 0.828 | 0.484 | 0.671 | normal |
|  | 1 | 0.869 | 0.518 | 0.698 | normal |
|  | 1.5 | 0.897 | 0.535 | 0.721 | normal |
| Retrohippocampal | 0.1 | 0.213 | 0.181 | 0.113 | failed |
|  | 0.25 | 0.218 | 0.174 | 0.132 | with holes |
|  | **0.5** | **0.207** | **0.159** | **0.132** | **normal** |
|  | 0.75 | 0.211 | 0.160 | 0.138 | normal |
|  | 1 | 0.212 | 0.158 | 0.141 | normal |
|  | 1.5 | 0.210 | 0.156 | 0.140 | normal |
| Striatum | 0.1 | 0.204 | 0.195 | 0.060 | failed |
|  | 0.25 | 0.245 | 0.191 | 0.154 | with holes |
|  | **0.5** | **0.174** | **0.142** | **0.101** | **normal** |
|  | 0.75 | 0.175 | 0.141 | 0.105 | normal |
|  | 1 | 0.177 | 0.142 | 0.107 | normal |
|  | 1.5 | 0.179 | 0.143 | 0.108 | normal |
| Fiber tracts | 0.1 | 0.096 | 0.083 | 0.049 | failed |
|  | 0.25 | 0.124 | 0.107 | 0.061 | with holes |
|  | **0.5** | **0.127** | **0.108** | **0.066** | **normal** |
|  | 0.75 | 0.136 | 0.114 | 0.074 | normal |
|  | 1 | 0.141 | 0.118 | 0.077 | normal |
|  | 1.5 | 0.144 | 0.119 | 0.082 | normal |
| Ventricular | 0.1 | NaN | NaN | NaN | failed |
|  | 0.25 | 0.132 | 0.116 | 0.062 | with holes |
|  | 0.5 | 0.114 | 0.100 | 0.054 | normal |
|  | 0.75 | 0.103 | 0.092 | 0.047 | normal |
|  | 1 | 0.098 | 0.086 | 0.048 | normal |
|  | **1.5** | **0.096** | **0.084** | **0.048** | **normal** |


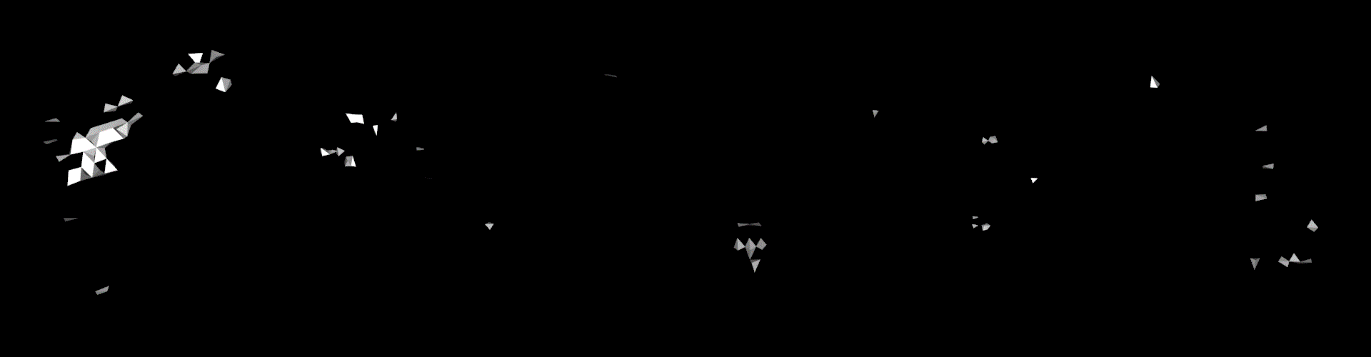


α=0.1


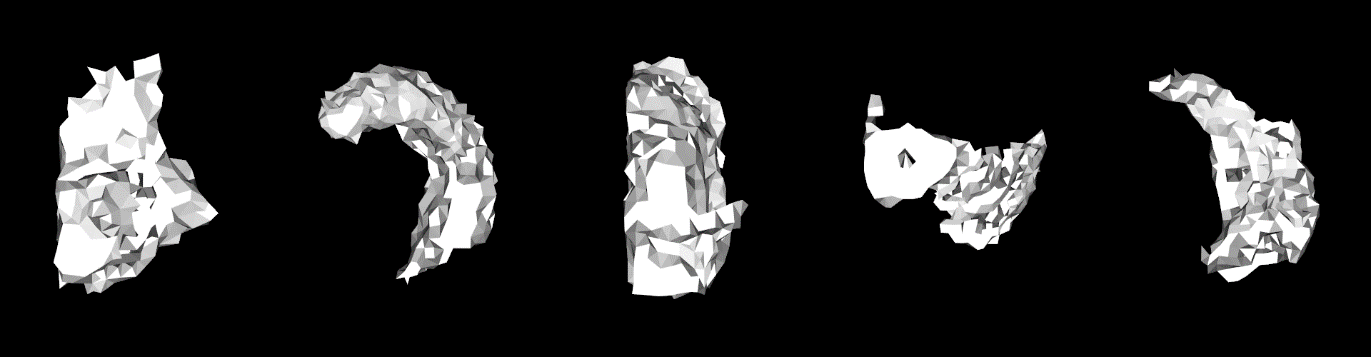


α=0.25


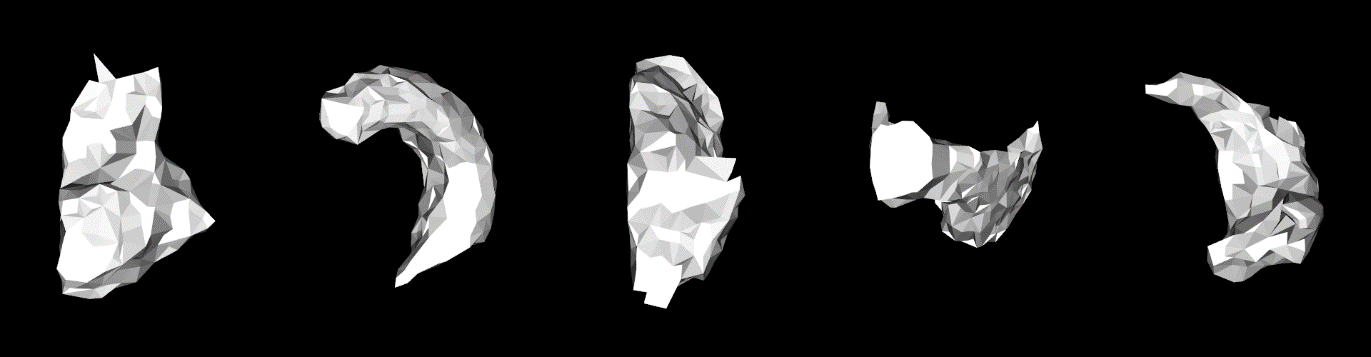


α=0.5


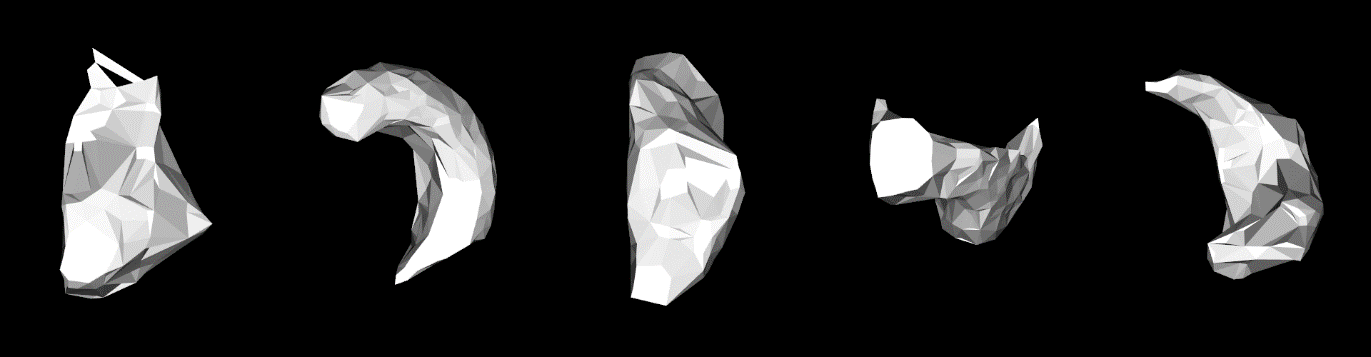

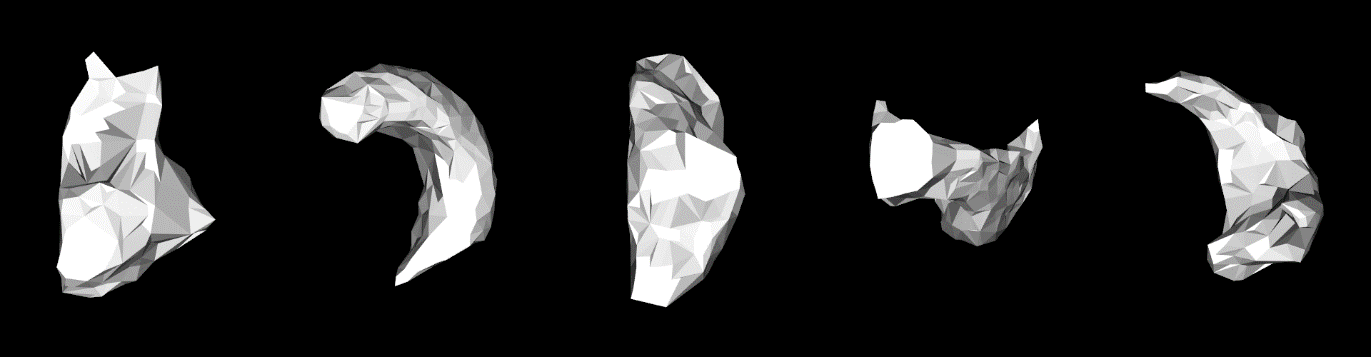


α=1

α=0.75


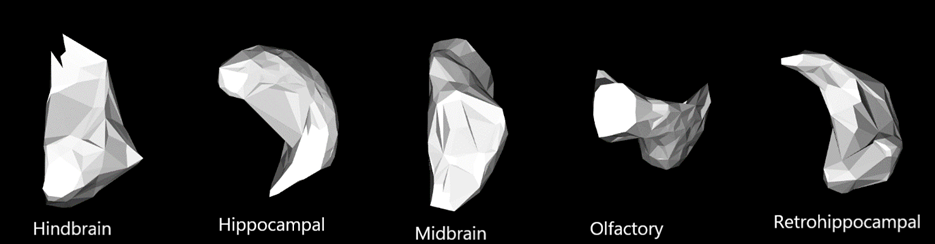


α=1.5

Figure SM5. Comparative Analysis of 3D Reconstructions with Varied Alpha Values.

Fig. SM5 provides a qualitative analysis of the impact of different alpha values in the Alpha Shape algorithm. When alpha is set to 0.1, the reconstruction result comprises a few small triangles due to the inadequate length of the ball radius to construct a full triangle. Consequently, only a few points can be reconstructed. When alpha is set to 0.25, the surface of the reconstruction is rough and features a few small holes, as depicted in Fig. SM6. However, at an alpha value of 0.5, Alpha Shape produces a better 3D reconstruction result with no visible small holes. If alpha is increased further, the reconstruction result begins to resemble that of the Hull Convex Algorithm, resulting in a surface that is too smooth and lacks concavity. Based on our quantitative and qualitative analysis, we have set the default alpha value to 0.5. Users can adjust the alpha value as needed in SMDB.


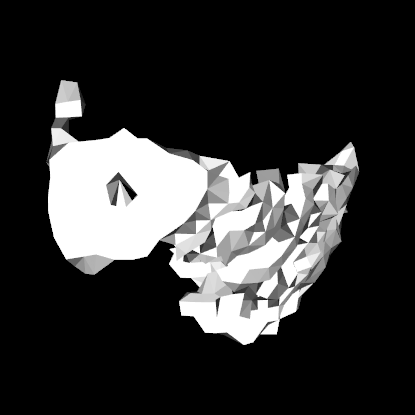


hole


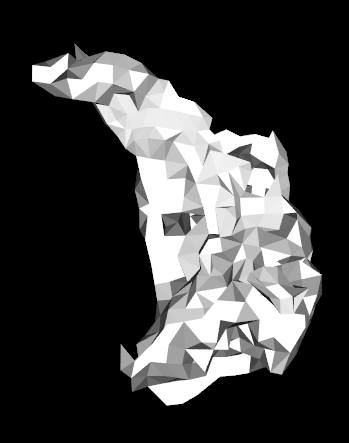


hole

Figure SM6. Holes Observed in the 3D Reconstruction with α=0.5.

### DBSCAN ablation analysis

We also compared the 3D reconstruction results with and without DBSCAN. Table 4 shows that the reconstruction results with DBSCAN outperform those without DBSCAN. Fig. SM7 provides a visual comparison of the two results, demonstrating that the results with DBSCAN are closer to the reference. As a result, we utilize DBSCAN to remove noisy data before conducting the reconstruction.


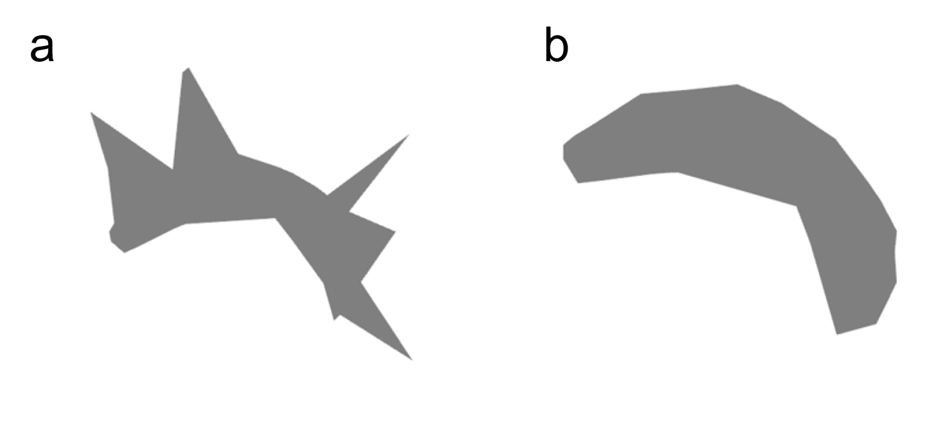


Figure SM7. Comparison of 3D reconstruction result without (a) or with (b) DBSCAN

Table SM4. Ablation. Comparison of 3D reconstruction result with or without DBSCAN

|  | with DBSCAN | | | without DBSCAN | | |
| --- | --- | --- | --- | --- | --- | --- |
| Dataset | RMSE | AED | SDED | RMSE | AED | SDED |
| Cerebellum | 0.113 | 0.102 | 0.049 | 0.122 | 0.111 | 0.051 |
| Cortical subplate | 0.254 | 0.202 | 0.154 | 0.260 | 0.209 | 0.155 |
| Hindbrain | 0.535 | 0.330 | 0.422 | 0.296 | 0.221 | 0.197 |
| Hippocampal region | 0.194 | 0.124 | 0.149 | 0.113 | 0.100 | 0.054 |
| Hypothalamus | 0.595 | 0.404 | 0.444 | 0.238 | 0.193 | 0.157 |
| Isocortex | 0.664 | 0.388 | 0.549 | 0.269 | 0.186 | 0.224 |
| Midbrain | 0.545 | 0.350 | 0.421 | 0.322 | 0.236 | 0.224 |
| Olfactory areas | 0.606 | 0.355 | 0.495 | 0.327 | 0.245 | 0.226 |
| Pallidum | 0.773 | 0.440 | 0.636 | 0.767 | 0.431 | 0.635 |
| Retrohippocampal | 0.250 | 0.179 | 0.174 | 0.207 | 0.159 | 0.132 |
| Striatum | 0.256 | 0.178 | 0.184 | 0.174 | 0.142 | 0.101 |
| Fiber tracts | 0.187 | 0.133 | 0.131 | 0.127 | 0.108 | 0.066 |
| Ventricular | 0.164 | 0.118 | 0.114 | 0.114 | 0.100 | 0.054 |

## REFERENCES

1. Rui, Y., Zhou, Z., Cai, X. and Dong, L. (2022) A novel robust method for acoustic emission source location using DBSCAN principle. *Measurement*, **191**.

2. Bernardini, F., Mittleman, J., Rushmeier, H., Silva, C. and Taubin, G. (1999) The ball-pivoting algorithm for surface reconstruction. *IEEE Transactions on Visualization and Computer Graphics*, **5**, 349-359.

3. Graham, R.L. (1972) An efficient algorith for determining the convex hull of a finite planar set. *Information Processing Letters*, **1**, 132-133.

4. Kazhdan, M., Bolitho, M. and Hoppe, H. (2006) Poisson Surface Reconstruction. *Eurographics Symposium on Geometry Processing*, 61-70.

5. Edelsbrunner, H., Kirkpatrick, D. and Seidel, R. (1983) On the shape of a set of points in the plane. *IEEE Transactions on Information Theory*, **29**, 551-559.

6. Taubin, G., Zhang, T. and Golub, G. (1996), *Computer Vision — ECCV '96*, pp. 283-292.

7. Wang, Q., Ding, S.L., Li, Y., Royall, J., Feng, D., Lesnar, P., Graddis, N., Naeemi, M., Facer, B., Ho, A. *et al.* (2020) The Allen Mouse Brain Common Coordinate Framework: A 3D Reference Atlas. *Cell*, **181**, 936-953 e920.

8. Ortiz, C., Navarro, J.F., Jurek, A., Martin, A., Lundeberg, J. and Meletis, K. (2020) Molecular atlas of the adult mouse brain. *Sci Adv*, **6**, eabb3446.
