# Supplementary material for: SMDB: a Spatial Multimodal Data Browser
Source: Nucleic Acids Res. 2023 May 22;51(W1):W553–9. doi: 10.1093/nar/gkad413 (PMC10320082; doi:10.1093/nar/gkad413)
Supplement: gkad413_Supplemental_Files [file gkad413_supplemental_files.zip › Supplementary_Tables.docx]

| Region | GeneName | pvalue | log2FC | pct.1 | pct.2 | adj.pvalue | Reference |
| --- | --- | --- | --- | --- | --- | --- | --- |
| DLS | Rgs4 | 8.97E-24 | 0.4682658 | 0.989 | 0.962 | 1.38E-19 |  |
|  | Coch | 1.89E-12 | 0.4558337 | 0.671 | 0.484 | 2.90E-08 | Märtin, Antje et al. (2019). |
|  | Gpr155 | 6.74E-09 | 0.3796088 | 0.848 | 0.738 | 1.03E-04 | Märtin, Antje et al. (2019). |
|  | Nefm | 4.88E-10 | 0.3738381 | 0.789 | 0.65 | 7.48E-06 | Märtin, Antje et al. (2019). |
|  | Kcnk2 | 1.16E-08 | 0.3480484 | 0.879 | 0.799 | 1.78E-04 | Djillani, Alaeddine et al. (2019). |
|  | Acvrl1 | 6.62E-08 | 0.2829003 | 0.303 | 0.143 | 1.01E-03 |  |
|  | Cnr1 | 7.25E-62 | 1.006155 | 0.964 | 0.848 | 1.11E-57 | Märtin, Antje et al. (2019). |
| DMS | Crym | 2.26E-70 | -1.2480313 | 0.655 | 0.965 | 3.46E-66 | Märtin, Antje et al. (2019). |
|  | Calb1 | 3.04E-15 | -0.486534 | 0.915 | 0.953 | 4.66E-11 | Pan, Jing et al. (2019). |
|  | Pdyn | 1.41E-06 | -0.4706257 | 0.669 | 0.714 | 2.16E-02 | Brimblecombe, Katherine R & Stephanie J Cragg (2017). |
|  | Ppp1r2 | 7.50E-23 | -0.4619236 | 0.913 | 0.965 | 1.15E-18 |  |
|  | Gda | 9.60E-09 | -0.3386631 | 0.829 | 0.883 | 1.47E-04 |  |
|  | Id4 | 2.14E-09 | -0.3273889 | 0.621 | 0.77 | 3.28E-05 | Stanley, Geoffrey et al. (2020) |
|  | Hpcal4 | 1.85E-13 | -0.3190083 | 0.933 | 0.968 | 2.84E-09 |  |

Supplementary Table1. the differential expression genes of DLS and DMS reconstructed morphological regions.

Supplementary Table2. links and reference to the visualization tools

| tools | url | Reference |
| --- | --- | --- |
| Giotto Viewer | http://www.spatialgiotto.com | Dries, Ruben et al. (2021) |
| SpatialLIBD | https://bioconductor.org/packages/spatialLIBD | Pardo, Brenda et al. (2022) |
| STUtility | https://ludvigla.github.io/STUtility_web_site | Bergenstråhle, Joseph et al. (2020) |
| ST Viewer | https://github.com/jfnavarro/st_viewer |  |
| Cirrocumulus | https://cirrocumulus.readthedocs.io |  |
